# Life expectancy inequalities between regions of China 2004–2020: contribution of age- and cause-specific mortality

**DOI:** 10.3389/fpubh.2023.1271469

**Published:** 2023-12-18

**Authors:** Leyi Zhang, Lijuan Sun

**Affiliations:** ^1^School of Insurance, University of International Business and Economics, Beijing, China; ^2^School of Mathematics, Baotou Teachers' College, Baotou, China

**Keywords:** life expectancy, health disparities, epidemiological transition, mortality improvement, health inequalities, regions of China

## Abstract

**Background:**

China's rapid economic and social development since the early 2000s has caused significant shifts in its epidemiological transition, potentially leading to health disparities across regions.

**Objectives:**

This study employs Life Expectancy (LE) to assess health disparities and trends among China's eastern, central, and western regions. It also examines the pace of LE gains relative to empirical trends and investigates age and causes of death mortality improvement contributing to regional LE gaps.

**Data and methods:**

Using a log-quadratic model, the study estimates LE in China and its regions from 2004 to 2020, using census and death cause surveillance data. It also utilizes the Human Mortality Database (HMD) and the LE gains by LE level approach to analyze China and its regions' LE gains in comparison to empirical trend of developed countries. The study investigates changes in LE gaps due to age and causes of death mortality improvements during two periods, 2004–2012 and 2012–2020, through the LE factor decomposition method.

**Results:**

From 2000 to 2020, China's LE exhibited faster pace of gains compared to developed countries. While men's LE growth gradually aligns with empirical trends, women experience slightly higher growth rates. Regional LE disparities significantly reduced from 2004 to 2012, with a marginal reduction from 2012 to 2020. In the latter period, the changing LE gap aligns with expected trends in developed countries, with all Chinese regions surpassing empirical estimates. Cardiovascular diseases and malignant neoplasms emerged as the primary contributors to expanding regional LE gaps, with neurological disorders and diabetes playing an increasingly negative role.

**Conclusion:**

LE disparities in China have consistently decreased, although at a slower pace in recent years, mirroring empirical trends. To further reduce regional LE disparities, targeted efforts should focus on improving mortality rates related to cardiovascular diseases, neoplasms, neurological disorders and diabetes, especially in the western region. Effective health interventions should prioritize equalizing basic public health services nationwide.

## 1 Introduction

Orman's epidemiological transition theory (1) provides a comprehensive framework for understanding national-level mortality patterns and causes of death. It underscores the shift from infectious to chronic diseases as the primary determinants of morbidity and mortality, a transition largely completed in developed countries but still ongoing in developing countries (2). LE serves as a crucial health indicator, reflecting overall population mortality across age groups. Following the mortality transition, the gradual increase of LE can be divided to two phases: initially, slow growth in LE associated with the diffusion of improved hygiene and nutrition, followed by a period of accelerated improvement, especially in the mortality of infants and children, accompanied by interventions in public health and basic medical care. In the second phase, the increase in LE is primarily due to infectious diseases across age groups and chronic diseases at older ages. However, the more challenging improvement in mortality from chronic diseases results in a slower increase in LE (3, 4).

With China's rapid economic development, its epidemiological transition has been rapid since the late 1970s (5, 6), exhibiting a transition period shorter than many other countries (7). However, rapid socioeconomic growth has also resulted in significant regional variation, particularly in key determinants of health such as income, education, and access to medical services. Consequently, the health gap between regions in China may be undergoing rapid changes, giving rise to substantial health disparities among regions (8). Despite notable improvements in the health gap between the eastern and western regions over the past two decades—a significant reduction in the mortality gap among children under 5 years from 42 to 7‰ between 2000 and 2020—the health disparity remains significant (9). Examining the age-standardized mortality rate in 2020 reveals markedly elevated rates for all causes in the western region, which are 24 and 7% higher than those in the central and eastern regions. This trend is consistent across categories of death cause, including infectious diseases, maternal and infant diseases, chronic diseases, and injuries (10).

The existing literature contains numerous estimates of China's LE. However, there is a noticeable scarcity of comprehensive studies focusing on regional LE estimates within China. The extant estimates (11) primarily present results for individual years and lack detailed life tables, methodological descriptions, and in-depth analyses of trends and underlying causes for the observed regional disparities. Consequently, there is a pressing need to address this gap by delving into the estimation methodologies pertaining to regional mortality levels in China. Such efforts would entail a rigorous analysis of the evolving trends in regional LE disparities and an exploration of the underlying factors driving these disparities. Additionally, it is essential to propose targeted improvement measures aimed at reducing health inequality.

To address these objectives, this article adopts a multifaceted approach. Firstly, the log-quadratic model is employed to estimate LE in both China and its regions from 2004 to 2020. This estimation framework allows for an exploration of the changing trends in regional LE disparities. Secondly, by leveraging data from the HMD and comparing the LE gains by level of LE, an analysis is conducted to discern the pace of LE growth in China and its regions relative to empirical trends observed in developed countries. Thirdly, an investigation into the age and cause of death factors contributing to changes in LE gaps between regions in China is carried out using the factor decomposition method of LE. Finally, this study formulates health intervention policies designed to address the identified disparities and improve overall healthcare outcomes. These initiatives will make substantial contributions to the understanding of China's health landscape and the promotion of health equity.

## 2 Data and methods

### 2.1 Source of data and data classification

The data of LE in developed countries is from HMD (12). The HMD contains sex-disaggregated period and population life table estimation results of 41 countries or regions in recent decades, primarily developed countries in Europe and other regions, with high data quality and reliability. For the purposes of estimating and comparing, we selected 3029 sex-disaggregated life table results from 23 countries or regions in the HMD from 1970 to 2020. For specific selected countries, please refer to Supplementary Table S1.

The data utilized in this study to estimate LE in China is from multiple data sources. Firstly, the census data of China provides regional gender- and age-specific population data for the years 2000, 2010, and 2020 (13–15). Secondly, the datasets of statistics on women and children in China 2014 and 2020 (9, 16), contains reginal mortality rates of children under 5 years from year 1991 to 2020. Thirdly, the National Disease Surveillance Point system(DSPs) China death cause surveillance dataset form year 2004 to 2012 (17, 18), and the China death cause surveillance dataset form year 2013 to 2020 (10, 19), these datasets offer death registration data by gender, age, and cause of death.

The regional classification employed in this study follows the division method of the National Bureau of Statistics, which categorizes the regions into three regions: the eastern, central, and western region. For further details regarding the regional division, please refer to Supplementary Table S1. This region classification is also utilized within the death cause surveillance dataset.

The International Classification of Diseases, Tenth Revision (ICD-10), is utilized to classify diseases based on specific characteristics and coding methods. Since 2002, the statistics in the death cause surveillance dataset have adopted the ICD-10 standard. According to this standard, causes of death are divided into three broad categories: infectious, maternal and child, and nutritional deficiency diseases; chronic and non-communicable diseases; and injuries. These three categories are further subdivided into 21 sub-categories. For analysis purposes, a focused set of diseases is chosen based on those with a death count exceeding 1% of the total deaths. For specific selected subgroups, please refer to Supplementary Table S1. Specifically, disease categories, such as conditions originating in the perinatal period, congenital abnormalities, respiratory infections, and infectious and parasitic diseases, primarily affect maternal and child health.

### 2.2 Method of LE estimation

Given the known data quality issues in China's census and death registration data, particularly the problem of underreporting of deaths (20, 21), the traditional life table method has exhibited suboptimal performance in estimating LE. Consequently, the model life table method, as an indirect estimation approach, has gained prominence in estimating the LE of China. Two advanced model life table methods are modified Brass logit and log-quadratic model (22, 23). Notably, both the WHO and the IHME have employed the updated version of modified Brass logit model, which is called GBD relational model life table system, to estimate mortality levels globally in their respective GHE2019 and GBD2019 studies. These efforts have included LE estimates for China spanning the years 2000 to 2019.

However, for the purposes of this study, the GBD model life table system employed by WHO and IHME is deemed inapplicable. This arises from the challenges associated with accessing the data used in the IHME model, the limited description of its estimation methodology, and the intricacies of the IHME model concerning missing data imputation, model complexity, and computational demands, rendering it less accessible for replication, utilization, and result-data interpretation (24). Nevertheless, the techniques used for parameter adjustment in GHE2019 and GBD2019 are worth learning from.

In this study, we adopt the log-quadratic model (23) for estimating LE of China and its regions. This choice is informed by the model's interpretability, flexibility, and ease of application, also it has same accuracy with the GBD model (23). However, one critical consideration in the application of log-quadratic model is whether the model can accurately capture the characteristics of the specific population (25). Research conducted by Zhang et al. (26) has affirmed the suitability of this model for China's data and its ability to faithfully represent the age-specific mortality patterns observed in China. Thus, we posit that the log-quadratic model stands as the optimal choice for this study.

### 2.3 Data handling and quality control measures

Typically, the log-quadratic model employs _5_q_0_ and _45_q_15_ as parameters for mortality estimation. The _5_q_0_ is employed to access the overall mortality level, while _45_q_15_ describes the magnitude and direction of the observed deviation of the mortality pattern among adults from the standard mortality pattern (27). It is required to adjust these parameters prior to LE estimation. Since the assumption of population closure is not upheld, the conventional Death Distribution Method is not a suitable method to estimate the completeness of adult death registration in China's regional DSPs and census data. Instead, the empirical model proposed by Adair and Lopez (28) is not confined by the assumption and, therefore, proves to be a more suitable alternative for our estimation needs.

To estimate the _5_q_0_ for the regions from 2004 to 2020, we employed the penalized smoothing spline method (29) to smooth _5_q_0_ data. The estimation of adult mortality involves a four-step process. First, using the population and death data from the death surveillance datasets and the censuses, the crude central death rate for adults aged 15–59 is calculated. Second, the method proposed by Greville (30) is applied to convert the crude central death rate into crude mortality rate. Third, an empirical model (28) is used to estimate the completeness of adult death registration, and the crude mortality rate is adjusted by the result. Fourth, the adjusted adult mortality rate is smoothed using the penalized smoothing spline method from 2004 to 2020.

Moreover, to ascertain the effectiveness of the LE estimation method employed in this study, we intend to compare our estimates of China's LE with those produced by WHO, IHME, and the China Statistics Yearbook.

### 2.4 Method of LE gains by level of LE

To compare the changing trend of LE between China and developed countries, we utilize the method of comparing LE gains by level of LE, as employed in the World Population Prospects 2019 (31). We believe this method better reflects the pace of LE gains in China by comparing it to the growth rate of developed countries at the same LE level. Since each country is in a different stage of mortality transition, the pace of LE gains varies. We are also comparing the LE gains in the regions of China with the empirical LE gains in developed countries. Additionally, we apply the penalized smoothing splines method to estimate the empirical process of LE gains for countries in the HMD. This method is preferred over the double logistic function, as we are specifically comparing LE gains in the second stage of LE gains.

### 2.5 Method of LE decomposition

The Arriaga's LE decomposition method (32) is employed to analyze the LE change in the regions of China. This method enables us to trace the underlying age and cause factors that lead to the changing trend of LE disparities at the regional level. The decomposition results can also provide evidence for making health-related suggestions to reduce LE disparities. For calculation, suppose *x* represents the age group, and *t* denotes time point, mortality is represented by the age-specific mortality ratecorresponding to the abbreviated life table with LE at birth *e*_0_(*t*_1_). After, the LE at birth reaches, and the corresponding age-specific mortality rate is *m*_*x*_(*t*_2_). The changes in LE can be decomposed into the sum of age-specific and cause-specific LE, and calculated as:


(1) $$e_{\text{0}}(t_{2})-e_{\text{0}}(t_{1})=\sum_{x}{\;}_{n}\Delta_{x}\text{=}\sum_{x}\sum_{i}{\;}_{n}\Delta_{x}^{i}$$

where *n Δ*_*x*_ and $n\;\Delta_{x}^{i}$ denotes the contribution of all-cause and causemortality improvement to the change of LE. Further, the contribution of age- and cause-specific mortality to the change of LE can be calculated as:


(2) $$\begin{array}{l} n^{\;\Delta_{x}^{i}}\;=\;n^{\;\Delta_{x}}\;\cdot \;\frac{n^{\;m_{x}^{i}\left(t_{2}\right)}\;-\;n^{\;m_{x}^{i}\left(t_{1}\right)}}{n^{\;m_{x}\left(t_{2}\right)}\;-\;n^{\;m_{x}\left(t_{1}\right)}} \end{array}$$

To compare the changing trends in regional LE gains, we divide the period into two parts: 2004–2012 and 2012–2020.

### 2.6 Tools used for analysis

We used R software (version 3.4.1) for calculations. Specifically, we used the MortCast package's logquad function to estimate LE, the smooth spline function in pspline package to perform penalized smoothing spline, and ggplot2 package to draw graph.

## 3 Results

### 3.1 Validation of LE estimation

Supplementary Table S2 shows estimates of adult death registration completeness, _5_q_0_ and _45_q_15_ of China and regional of China. Supplementary Table S2 reveals lower completeness observed in the western regions and higher completeness in the eastern regions. Over the years, the completeness has shown an increasing trend, especially in central and western regions. In 2020, the completeness reached 98.3 and 95.5% for males, and 96.5 and 90.6% for females in the eastern and western regions, respectively.

Supplementary Table S3 compares the estimates of _5_q_0_ and _45_q_15_ by WHO, IHME, and this study. The results indicate that the _5_q_0_ estimates from this study are very close to those of WHO, and the _45_q_15_estimates are close to those of IHME. This demonstrates the effectiveness of our estimation for these two parameters.

**Figure 1:**
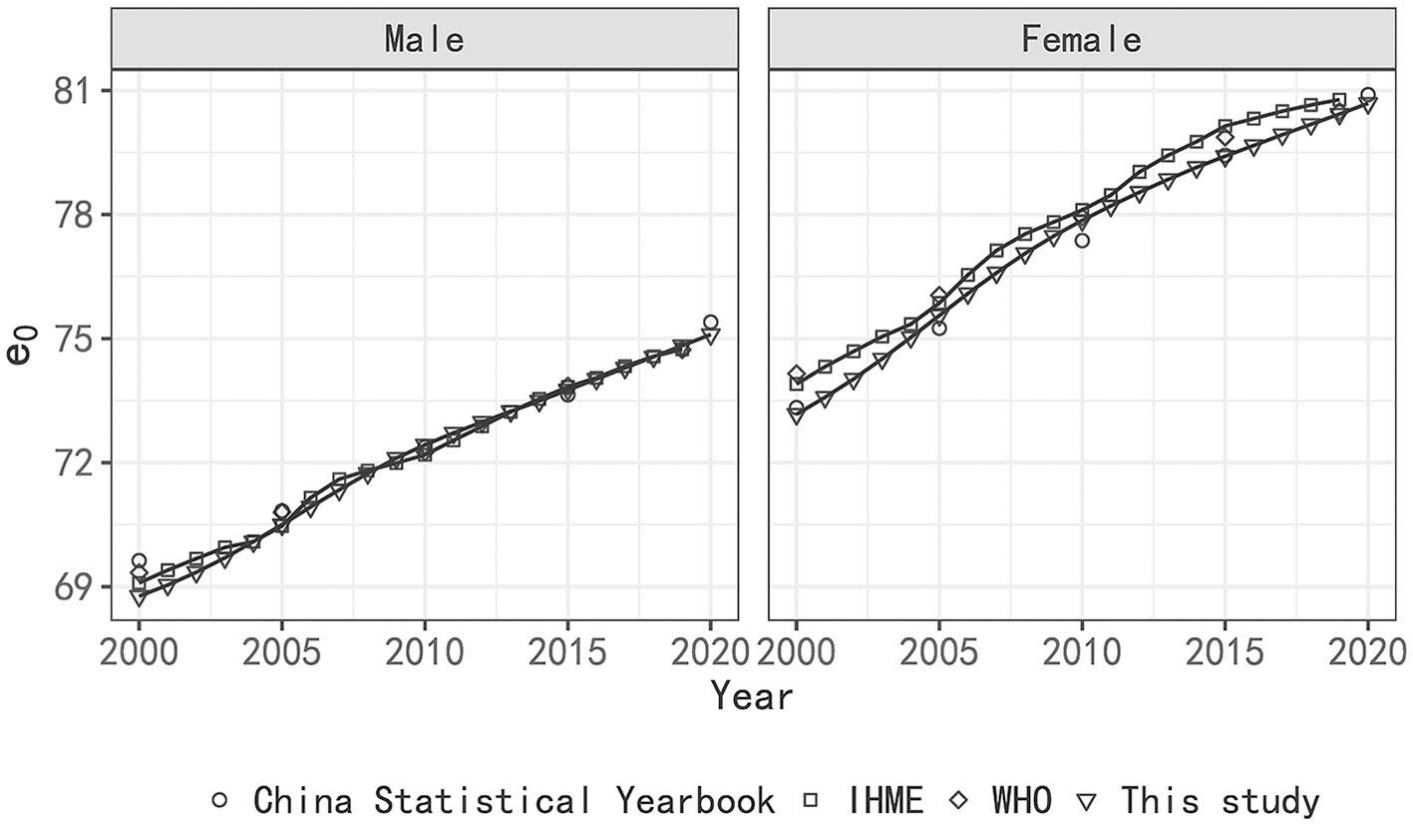
LE estimation of China 2000–2020 by WHO, IHME, China Statistical Yearbook and this study.

The estimated LE of China and its regions are presented in Figure 1 and Supplementary Table S4. Figure 1 presents the results of the LE estimation of China from 2000 to 2020. The applicability of the log-quadratic model to Chinese data was verified by comparing the estimated LE of China with those of the IHME, WHO, and China Statistics Yearbook (33–35). As shown in Figure 1, the estimated results of this study were very close to those of the other institutions, except for the estimation of men in 2000, which was slightly smaller. This consistency in the estimated results indicates the applicability of the log-quadratic model to the estimation of regional LE of China.

### 3.2 Empirical evidence of LE gains

**Figure 2:**
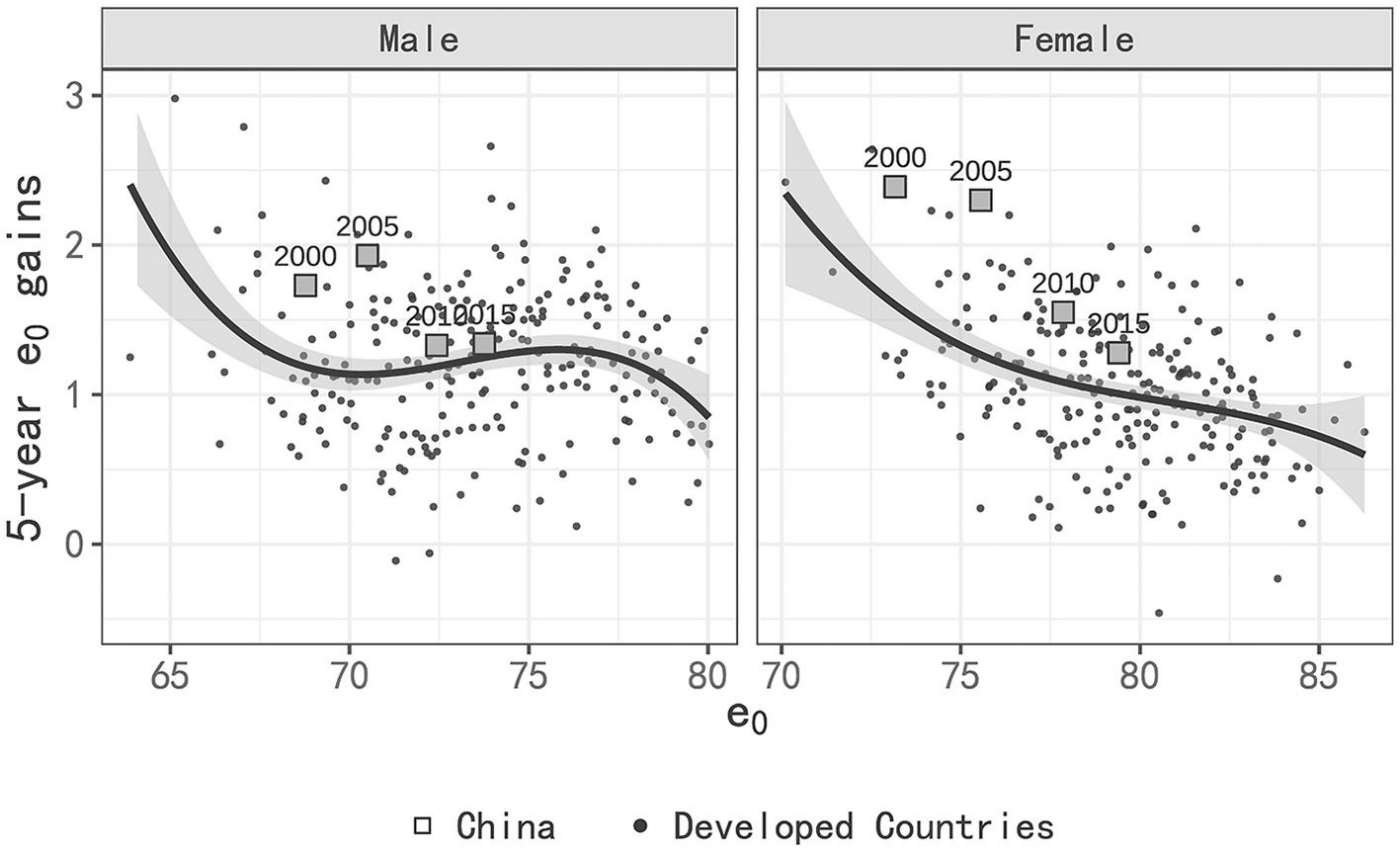
Five-year LE gains by level of LE of China in compared with developed countries.

Figure 2 presents the 5-year gains of LE in China and developed countries by level of LE. Figure 2 shows that the gains of male LE in China have decreased from a much higher than that of developed countries to being slightly higher, while for females, the gains have also declined sharply but still remain higher than those in developed countries. As shown in Figure 2, the LE gap between China and developed countries decreased rapidly from 2000 to 2010 and slowed in next 10 years for both males and females.

Regarding the pace of gains in LE, while males in China initially experienced more rapid gains compared to developed countries, their pace of gains has now slowed down, leaving them with only a slight lead. Similarly, the pace of gains for females in China has decreased significantly, but they still have higher gains in LE compared to those in developed countries. During 2015–2020, male LE in China increased by 0.1 years more than that of developed countries, while female LE increased by 0.3 years more than that of developed countries.

### 3.3 LE disparity between regions of China

**Figure 3:**
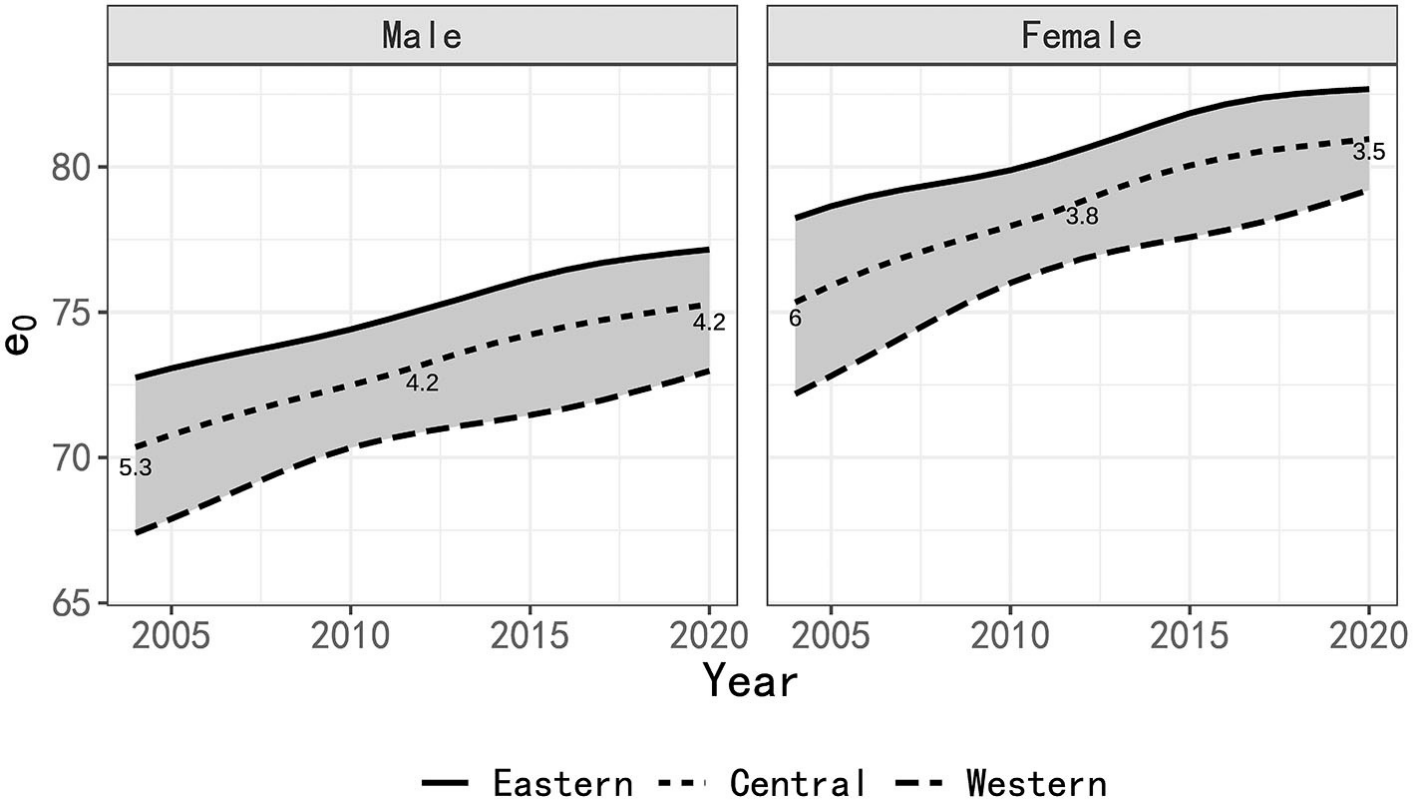
LE estimation in regions of China 2004–2020.

Figure 3 visually elucidates the overarching upward trajectory of LE across all regions spanning the years 2004 to 2020. Notably, the LE differential between the eastern and western regions initially exhibited a decreasing trend, which was subsequently followed by a modest reduction. Among males, the disparity expanded from 5.3 years in 2004 to 4.2 years in 2012, eventually stabilizing at 4.2 years in 2020. Likewise, among females, the disparity dwindled from 6 years in 2004 to 3.8 years in 2012, and further exhibited a marginal reduction to 3.5 years in 2020.

The time frame between 2004 and 2012 witnessed noteworthy improvements in male and female mortality rates within the western region, contributing increments of 3.4 years and 4.6 years, respectively. These increments surpassed the corresponding gains observed in the eastern region (2.4 and 2.5 years) and the central region (2.9 and 3.6 years). Consequently, a substantial narrowing of the LE gap between the eastern and western regions was evident during this period.

However, the period from 2012 to 2020 witnessed a shift in LE dynamics. The increase in LE for males in both the western and eastern regions diminished to 2.1 years. Among females, the LE increment in the western region regressed to 2.4 years, while it surged by 2.1 years in the eastern region. Consequently, the LE disparity between these two regions exhibited a marginal reduction.

Based on the empirical estimates of 5-year LE gains in developed countries in Figure 2, we can calculate that the LE gains in the eastern and western regions from 2012 to 2020 should be 2.07 years and 1.86 years, respectively (adjusted proportionally to an 8-year period). This would suggest an estimated reduction in the LE gap of 0.19 years. However, it's important to note that the actual LE gap between the two regions remains unchanged. The reason for this is that the actual increase of 2.1 years in the western region is higher than the empirical estimate of 1.86 years, and the estimated value in the eastern region is close to the actual value.

For women, following the patterns observed in Figure 2, it is estimated that the LE growth in the eastern and western regions from 2012 to 2020 should be 1.77 years and 1.48 years, respectively. This suggests a reduction in the gap by 0.29 years, which indeed aligns with the actual reduction of 0.3 years. The numerical changes are relatively consistent. Furthermore, it's worth noting that the growth of LE in the eastern, central, and western regions is significantly faster than the empirical estimate, similar to the changes in national LE growth relative to the empirical estimate discussed in Section 3.2.

### 3.4 LE decomposition by age

**Figure 4:**
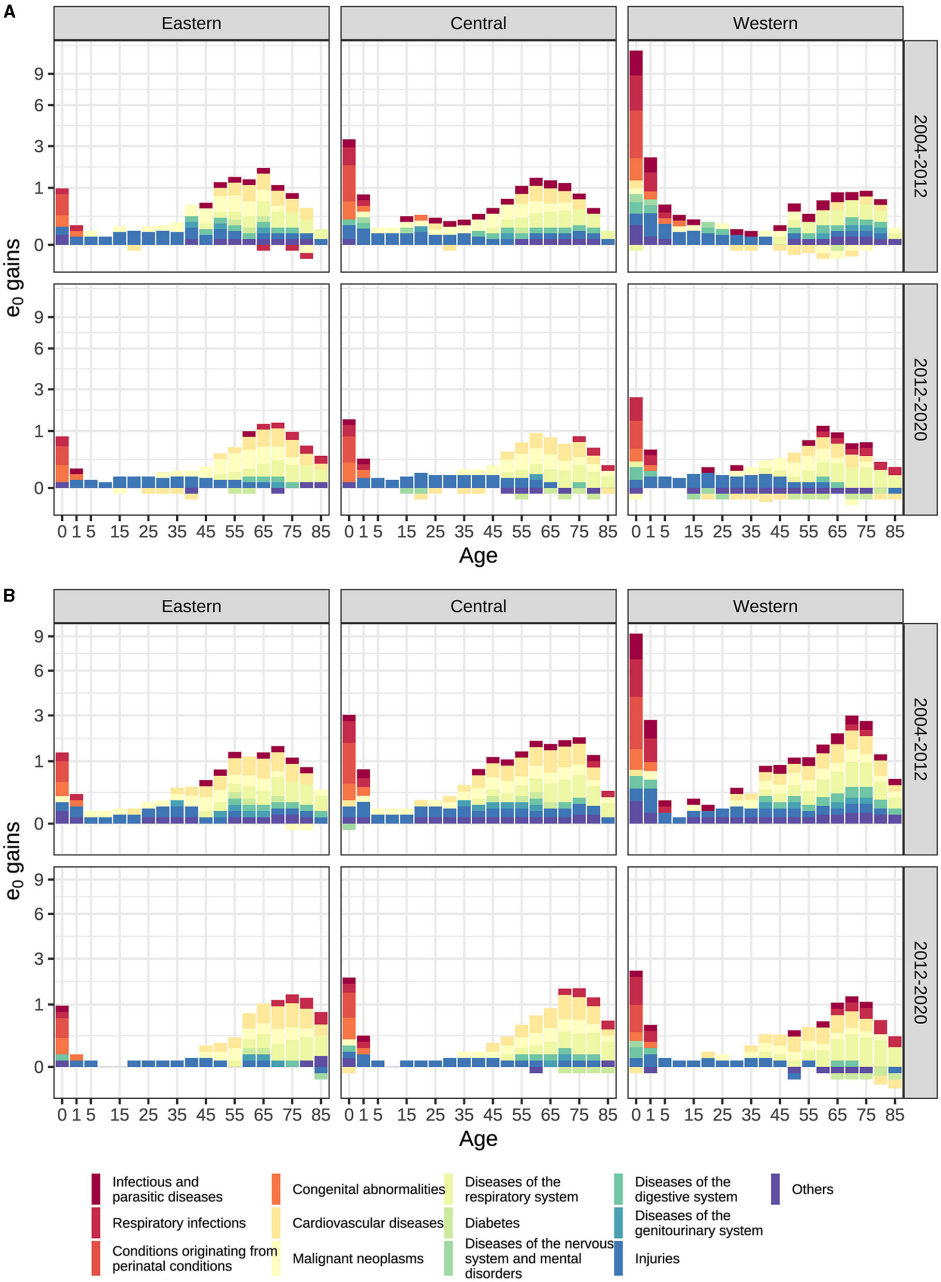
**(A**) Male **(B)** Female LE gains age- and cause-specific decomposition by regions of China (The proportions of the y-axis have been altered and the gains in LE are rounded to the nearest 0.1 year in order to enhance readability).

Figure 4 presents the result of age- and cause- specific decomposition for changes in LE in regions of China 2004–2020, for detail data of LE age specific decomposition please refer to Supplementary Table S5. From 2004 to 2012, the narrowing of the LE disparity can primarily be attributed to improvement in child mortality. Specifically, the mortality improvements among male children under the age of 5 in the western region contributed 2.2 years and 0.7 years to their LE gains, surpassing the contributions observed in the eastern and central regions (0.9 years and 0.3 years, respectively). Conversely, contributions from individuals aged over 60 years were 0.7 years in the western region, which is slightly less than the corresponding figures of 1 year in the eastern and central regions. This trend was echoed among females. Moreover, a noteworthy negative contribution was observed among males aged 30–54 in the western region.

In the subsequent period spanning 2012 to 2020, the contribution of mortality improvements among children under 5 years declined significantly to 0.7 years in the western region, in comparison to 0.5 years and 0.3 years for the central and eastern regions, respectively. The contributions stemming from individuals aged 60 and over were 0.8 years in the western region, slightly less than the figures of 1 year and 1.2 years for the central and eastern regions, respectively. This pattern was consistent among females as well. Additionally, negative contributions were observed among males aged over 15 in all three regions and among females aged over 60 in the central and western regions. During this period, the LE gains in all three regions predominantly emanated from mortality improvements among the older adult.

### 3.5 LE decomposition by cause

Figure 4 presents the result of age and cause-specific decomposition of male and female LE changes in regions of China. For the contribution of specific death cause mortality improvement to regional LE change, please refer to Supplementary Table S6.

Both Figure 4 and Supplementary Table S6 reveal, from 2004 to 2012, those regional variations in the contribution of infectious diseases and maternal and child diseases were primary factors driving the reduction of the LE gap between regions in China. Specifically, conditions in the perinatal period, respiratory infections, infectious and parasitic diseases contributed to a combined reduction of 1.43 years for male and 1.48 years for female. The expansion of the male LE gap was primarily influenced by cardiovascular disease and malignant neoplasms, contributing 0.77 years. In contrast, the expansion of the female LE gap was impacted to a lesser extent by diabetes and cardiovascular disease, contributing 0.07 years. Notably, the western region exhibited a significantly higher contribution in terms of improving the mortality rates associated with infectious diseases and maternal and child disease, as compared to the central and eastern regions, but lower in chronic diseases. Specifically, negative contributions to LE in diabetes and cardiovascular disease have emerged among men in the western regions, especially age 30–54. Furthermore, diseases in other categories also contribute to the reduction of LE, particularly in children aged 0–4 years, primarily due to disparities in the mortality improvement of nutritional deficiency diseases.

From 2012 to 2020, infectious diseases, maternal and child diseases continued to contribute to the reduction of regional LE gaps but with a significant decline compared to the previous period. Conversely, chronic diseases contributed to the widening of regional LE gaps. Specifically, the diseases of the respiratory system, respiratory infections, and injuries accounted for a decrease of 0.58 years in males and 0.74 years in females. Conversely, cardiovascular disease and malignant neoplasms were responsible for an increase of up to 0.49 years in males and 0.32 years in females. The negative contribution of diseases of the nervous system and mental disorders, diabetes and disease in other category further deepen in compare with last period. The western region has the highest negative contribution related to these diseases, while the eastern region demonstrated the smallest negative contribution. Regarding age distribution, the negative contribution of diabetes primarily affects males over 40 and the older adult of female over 70, the nervous system and mental disorders concentrated among males aged 5–29, whereas the cardiovascular disease in men mainly occurs between the aged 30–44. Other diseases including genitourinary system also contributed to the expansion, albeit with a relatively smaller impact on LE. Tumors excluding malignant neoplasms in other categories exhibit a negative contribution to western region and the widening gap, particularly among men over 50 years old and women over 65 years old. Also, nutritional deficiency diseases in other categories impact the expanding gap among the older adult over 85 years old, leading to increased mortality rates in the western region and decreased rates in the eastern region.

## 4 Discussion

Since the inception of economic reforms and the policy of openness in China, the country has undergone a rapid epidemiological transition. Correspondingly, there has been a sustained and substantial increase in the LE of the Chinese population. Notably, LE rose from 67.8 years in 1981 to 71.4 years in 2000 (13). This upward trajectory has persisted since 2000, with both men and women experiencing a notable gain of 6.3 and 7.5 years in their LE during the period 2000–2020. Additionally, our analysis, which compares China's LE growth with that of developed countries at equivalent LE levels, reveals that China's LE growth rate in this period continues to outpace the experiences of developed countries with comparable LE levels. However, it is important to note that in recent years, the growth rate of male LE has moderated to align with the average level estimated based on the experiences of developed countries. In contrast, the growth rate of female LE remains relatively swift.

Drawing from epidemiological transition theory, it is conceivable that LE disparities may emerge within rapidly transitioning countries, necessitating focused research on regional health inequalities. Regrettably, limited studies have thus far delved into the subject of regional health disparities in China. To address this, our analysis is constrained by data availability and focuses on the years 2004–2020, scrutinizing two distinct periods, 2004–2012 and 2012–2020, to capture evolving LE trends.

Our analysis reveals that the gap in LE between regions in China exhibited a noteworthy reduction from 2004 to 2012. This reduction is chiefly attributed to improvements in mortality associated with infectious and parasitic diseases, respiratory infections, and conditions originating in the perinatal period. Conversely, the widening gap during this period is primarily attributed to improvement in mortality rates related to malignant tumors and cardiovascular diseases. Notably, LE experienced significant growth across all regions during this period, with the western region outpacing the central and eastern regions in terms of LE growth. Additionally, when compared to empirical trends, China's LE growth rate exceeded that of developed countries at same LE levels.

However, from 2012 to 2020, the reduction in the LE gap between regions exhibited a marginal decrease, remaining unchanged for men and decreasing by 0.3 years for women. The result shows this trend is consistent with empirical trends in LE changes of developed countries. Specifically, for men, according to the empirical trend, the increase in LE in the eastern region should be higher than that in the western region during this period, and the LE gap should be smaller. However, in fact, because the increase in the eastern region is close to the estimated value, while the western region is higher than the empirical value, the regional gap in male LE remains unchanged. For women, growth should have been relatively higher in the west, and actual increases were consistent with this expectation. Furthermore, increases were higher than empirical estimates in all regions, particularly among women, which is consistent with trends of country.

In this period, the results of LE decomposition show that the causes of death contributing to the slightly reducing LE gap remained largely consistent with the previous period. In addition to the previously noted factors, diseases of the respiratory system and injuries emerged as contributors to narrowing the gap. Moreover, the growth in LE observed in various Chinese regions from 2012 to 2020 was primarily driven by improved mortality among older adults. Notably, the western region exhibited a more significant negative contribution to improving LE due to mortality from neurological and mental disorders, diabetes, and other causes compared to the central and eastern regions. These findings elucidate the key factors underpinning the recent changes in the LE gap between regions.

In 2020, notable disparities in mortality rates between regions in China persist. Standardized mortality rates for cardiovascular diseases, respiratory diseases, diabetes and injuries in the western regions were 19, 154, 17, and 35% higher than those in the eastern region, respectively, while malignant neoplasms were 7% lower (10). These disparities underscore the imperative to enhance health outcomes across the western region by addressing mortality rates in all age groups, with a particular focus on major chronic diseases and those with rising mortality rates, such as stroke among young men, diabetes, and Alzheimer's disease among the older adults.

The substantial reduction in the mortality rate of infectious diseases and maternal and child diseases in China can be attributed to the provision of quality primary health services, including vaccination, water, sanitation, hygiene, and effective disease surveillance and response systems (36). Furthermore, many studies have emphasized the superiority of primary health care, including chronic disease early detection and management, in improving the prevalence and mortality of chronic diseases compared to medical services, highlighting its vital role in reducing regional health disparities (37). Consequently, the government of China should prioritize “health equalities” and strengthen the equalization of basic public health services as a vehicle to achieve universal health coverage in China (38), as this would effectively narrow the health gap between the regions.

Notably, our estimation results indicate a resurgence in the LE growth rate in the western region after 2016. The dynamics of LE gap changes between regions in the future necessitate continued scrutiny. Additionally, the global impact of the COVID-19 pandemic has led to a significant drop in LE in developed countries from 2019 to 2020 (39). Among them, LE in the United States has dropped significantly, reaching 1.8 years (40). In contrast, China's stringent epidemic prevention and control measures limited the impact of COVID-19, resulting in a less pronounced impact on LE in 2020. Further investigation into the influence of COVID-19 on LE disparities among Chinese regions remains a subject of future research, contingent on the availability of new data.

Compared with the United States, the gap in LE between regions in China is slightly larger. In 2020, the LE gap between regions in the U.S. is 3.4 years for men and 3.1 years for women (40), comparing the state averages of the South and Northeast regions. This difference is slightly wider than the gap observed in 2019 before COVID-19 but remains slightly smaller than the gap between regions in China. However, since there is greater unbalanced economic development within provinces of China, the LE gap at this level also requires further research.

In summary, while China has made considerable strides in reducing regional disparities in LE, recent years have seen a slightly slower rate of reduction. However, this trend is consistent with empirical trends in LE changes. To continue bridging the regional LE gap of China, future efforts should concentrate on addressing mortality rates associated with cardiovascular diseases, neoplasms, and diabetes, while also paying attention to infectious diseases and maternal and infant health, especially in the western region.

## 5 Conclusion

The disparity in LE among regions in China has consistently decreased, although the pace of this reduction has recently slowed. This trend is consistent with empirical trends in LE changes. To narrow the regional LE gap in the future, targeted efforts should prioritize the improvement of mortality rates associated with cardiovascular diseases, neoplasms, neurological disorders and diabetes, especially in the western region. Effective health interventions should focus on achieving equalization of basic public health services across the country.

## Data availability statement

The original contributions presented in the study are included in the article/Supplementary material, further inquiries can be directed to the corresponding author.

## Ethics statement

The ethical permission for this study is unnecessary as the data used is pre-existing, publicly available, fully anonymized, and devoid of any personal or sensitive information about individuals.

## Author contributions

LZ: Conceptualization, Data curation, Formal analysis, Investigation, Methodology, Software, Visualization, Writing–original draft, Writing–review & editing. LS: Funding acquisition, Project administration, Resources, Supervision, Validation, Writing–review & editing.

## References

[1] Omran AR. The epidemiological transition: a theory of the epidemiology of population change. Milbank Mem Fund Q. (1971) 49:509–38. 10.2307/3349375 5155251

[2] Santosa A Wall S Fottrell E Högberg U Byass P. The development and experience of epidemiological transition theory over four decades: a systematic review. Glob Health Action. (2014) 7:1–16. 10.3402/gha.v7.23574 PMC4038769 24848657

[3] Riley JC. Rising Life Expectancy: A Global History. Cambridge: Cambridge University Press (2001).

[4] Fogel RW. The Escape From Hunger and Premature Death, 1700-2100: Europe, America, and the Third World, Vol. 38. Cambridge: Cambridge University Press (2004).

[5] Yang G Wang Y Zeng Y Gao GF Liang X Zhou M. Rapid health transition in China, 1990–2010: findings from the global burden of disease study 2010. Lancet. (2013) 381:1987–2015. 10.1016/S0140-6736(13)61097-1 23746901 PMC7159289

[6] Zhao Z Kinfu Y. Mortality transition in East Asia. Asian Popul Stud. (2005) 1:3–30. 10.1080/17441730500124626

[7] Yang G Kong L Zhao W Wan X Zhai Y Chen LC. Emergence of chronic non-communicable diseases in China. Lancet. (2008) 372:1697–705. 10.1016/S0140-6736(08)61366-5 18930526

[8] Frenk J Bobadilla JL Stern C Frejka T Lozano R. Elements for a theory of the health transition. Health Trans Rev. (1991) 1:21–38. 10148802

[9] Department of Social, Science and Technology, and Cultural Statistics National Bureau of Statistics of China. Statistics on Women and Children in China 2020. Beijing: China Statistics Press (2021).

[10] The National Center for Chronic and Non-communicable Disease Control and Prevention, The Statistical Information Center of National Health Commission. China Death Cause Surveillance Data Set 2020. Beijing: China Science and Technology Press (2021).

[11] National Health Commission. China Health Statistics Yearbook 2021. Beijing: China Union Medical University Press (2021).

[12] Human Mortality Database. University of California, Berkeley and Max Planck Institute for Demographic Research. (2022). Available online at: www.mortality.org (accessed March 6, 2023).

[13] Office of the Leading Group of the State Council for the Seventh National Population Census. China Population Census Yearbook 2021. Beijing: China Statistics Press (2022).

[14] Population Census Office Under the State Council Department of Population and Employment Statistics National Bureau of Statistics, Department Department of Population and Employment Statistics, National Bureau of Statistics. Tabulation on the 2010 Population Census of the People Republic of China. Beijing: China Statistics Press (2012).

[15] Population Census Office under the State Council Department of Population, Department of Social, Science and Technology Statistics, National Bureau of Statistics. Tabulation on the 2000 Population Census of the People Republic of China. Beijing: China Statistics Press (2002).

[16] Department of Social, Science and Technology, and Cultural Statistics National Bureau of Statistics of China. Statistics on Women and Children in China 2014. Beijing: China Statistics Press (2014).

[17] The National Center for Chronic and Noncommunicable Disease Control and Prevention. National Disease Surveillance Point System China death cause surveillance data set 2004. Beijing: Military Medical Science Press; 2009.

[18] The National Center for Chronic and Non-communicable Disease Control and Prevention. National Disease Surveillance Point System China Death Cause Surveillance Data Set 2011. Beijing: People's Health Publisher (2013).

[19] The National Center for Chronic and Non-communicable Disease Control and Prevention. National Disease Surveillance Point System China Death Cause Surveillance Data Set 2012. Beijing: Popular Science Press (2013).

[20] Goodkind DM. China's missing children: the 2000 census underreporting surprise. Population Stu. (2004) 58:281–95. 10.1080/0032472042000272348 15513284

[21] Cai Y. China's new demographic reality: learning from the 2010 census. Popul Dev Rev. (2013) 39:371–96. 10.1111/j.1728-4457.2013.00608.x 25620818 PMC4302763

[22] Murray CJ Ferguson BD Lopez AD Guillot M Salomon JA Ahmad O. Modified logit life table system: principles, empirical validation, and application. Pop Stu. (2003) 57:165–82. 10.1080/0032472032000097083

[23] Wilmoth J Zureick S Canudas-Romo V Inoue M Sawyer C. A flexible two-dimensional mortality model for use in indirect estimation. Pop Stu. (2012) 66:1–28. 10.1080/00324728.2011.611411 22150635 PMC4046865

[24] Mathers CD. History of global burden of disease assessment at the World Health Organization. Arch Public Health. (2020) 78:1–13. 10.1186/s13690-020-00458-3 32850124 PMC7443850

[25] Ouédraogo S. Estimation of older adult mortality from imperfect data. Demogr Res. (2020) 43:1119–54. 10.4054/DemRes.2020.43.38

[26] Zhang Z Dai Z Yang J. Applicability of two-dimensional death model to China's population death pattern. Chin J Popul Sci. (2017) 1:81–91.

[27] Moultrie TA Dorrington RE Hill AG Hill K Timaeus IM Zaba B. Tools for Demographic Estimation. Paris: International Union for the Scientific Study of Population (2013).

[28] Adair T Lopez AD. Estimating the completeness of death registration: an empirical method. PLoS ONE. (2018) 13:e0197047. 10.1371/journal.pone.0197047 29847573 PMC5976169

[29] Heckman N Ramsay JO. Spline Smoothing With Model Based Penalties. New York, NY: McGill (1996).

[30] Greville TN. Mortality tables analyzed by cause of death. Record Am Inst Actuaries. (1948) 37:283–94.

[31] United Nations. World Population Prospects 2019. Vol (ST/ESA/SE A/424). London: Department of Economic and Social Affairs: Population Division (2019), 141.

[32] Arriaga EE. Measuring and explaining the change in life expectancies. Demography. (1984) 21:83–96. 10.2307/2061029 6714492

[33] Institute for Health Metrics and Evaluation. Life Expectancy at Birth (Years). (2022). Available online at: https://www.healthdata.org (accessed August 1, 2022).

[34] WHO. Life Expectancy at Birth (Years). (2022). Available online at: https://www.who.int/data/ (accessed September 1, 2022).

[35] National Bureau of Statistics of China. China Statistical Yearbook 2020. Beijing: China Statistics Press (2021).

[36] Wang L Wang Y Jin S Wu Z Chin DP Koplan JP. Emergence and control of infectious diseases in China. Lancet. (2008) 372:1598–605. 10.1016/S0140-6736(08)61365-3 18930534 PMC7138027

[37] Fang P Dong S Xiao J Liu C Feng X Wang Y. Regional inequality in health and its determinants: evidence from China. Health Policy. (2010) 94:14–25. 10.1016/j.healthpol.2009.08.002 19735959

[38] Yuan B Balabanova D Gao J Tang S Guo Y. Strengthening public health services to achieve universal health coverage in China. BMJ. (2019) 365:l2358. 10.1136/bmj.l2358 31227480 PMC6598722

[39] Islam N Jdanov DA Shkolnikov VM Khunti K Kawachi I White M. Effects of covid-19 pandemic on life expectancy and premature mortality in 2020: time series analysis in 37 countries. BMJ. (2021) 375:1–14. 10.1136/bmj-2021-066768 34732390 PMC8564739

[40] Tejada-Vera B Salant B Bastian B Arias E. US Life Expectancy by State and Sex for 2019. Hyattsville, MD: National Center for Health Statistics (2022).

